# Effects of caffeine, methylliberine, and theacrine on vigilance, marksmanship, and hemodynamic responses in tactical personnel: a double-blind, randomized, placebo-controlled trial

**DOI:** 10.1080/15502783.2022.2113339

**Published:** 2022-08-18

**Authors:** Harry P. Cintineo, Marissa L. Bello, Alexa J. Chandler, Thomas D. Cardaci, Bridget A. McFadden, Shawn M. Arent

**Affiliations:** a Department of Exercise Science, University of South Carolina, Columbia, SC, USA; b Department of Kinesiology, Mississippi State University, Mississippi State, MS, USA; c Department of Pathology, Microbiology, and Immunology, University of South Carolina School of Medicine, Columbia, SC, USA

**Keywords:** Sports nutrition, dietary supplements, stimulants, reaction time

## Abstract

**Background:**

Tactical athletes require fast reaction times (RT) along with high levels of vigilance and marksmanship performance. Caffeine has been shown to improve these measures but also results in increased blood pressure and jitteriness. Research on other purine alkaloids, such as methylliberine and theacrine, has suggested they do not increase blood pressure or jitteriness to the same extent, but their impact on tactical performance is unknown.

**Methods:**

A between-subjects, randomized, placebo-controlled design was used to test the effects of placebo (PLA), 300 mg caffeine (CAF), and a combination of 150 mg caffeine, 100 mg methylliberine, and 50 mg theacrine (CMT) on RT and marksmanship along with hemodynamic and arousal measures following a sustained vigilance task in tactical personnel (n = 48). Following consumption of the supplement, participants underwent a 150-min protocol consisting of two rounds. Each round began with leisurely reading followed by a 30-min vigilance task before beginning two trials of movement and marksmanship tasks. Hemodynamics and felt arousal were assessed throughout the protocol. Composite Z-scores were calculated for overall performance measures at each timepoint, and mixed-effects models were used to assess differences in RT, accuracy, and composite Z-scores along with hemodynamics and felt arousal. An α-level of 0.05 was used to determine statistical significance, and Cohen’s *d* was used to quantify effect sizes.

**Results:**

A Group-by-Time interaction for vigilance RT (P = 0.038) indicated improvements for both CAF and CMT from round 1 to round 2 (P < 0.01) while PLA did not change (P = 0.27). No Group main effects or Group-by-Time interactions were found for movement or marksmanship performance (P > 0.20). Group main effects for systolic (SBP; P = 0.001) and diastolic blood pressure (DBP; P = 0.028) indicated higher SBP in CAF (P = 0.003, *d*= 0.84) and CMT (P = 0.007, *d*= 0.79) compared to PLA but only higher DBP in CAF (P = 0.025, *d*= 0.74). No Group-by-Time interaction or Group main effect was found for felt arousal (P > 0.16).

**Conclusions:**

These findings suggest similar benefits on RT during a vigilance task between CAF, containing 300 mg caffeine, and CMT above PLA, though CAF resulted in slightly less favorable hemodynamic changes. This study is the first to provide data showing similar efficacy of combined caffeine, methylliberine, and theacrine compared to double the caffeine dose consumed alone on vigilance RT but without a significant rise in DBP above PLA in tactical personnel.

## 1. Background

Military and law enforcement personnel are required to possess sufficient physical and cognitive fitness to perform their official duties. As such, these populations have been deemed ‘tactical athletes’ to emphasize these requirements. In a narrative review, Scofield and Kardouni [1] highlighted that tactical athletes must also have fast reaction times (RT), high levels of vigilance, defined as the ability to maintain alertness during extended periods, and proficient marksmanship capabilities. Most importantly, the physical fitness and performance parameters have an effect on mission success in these populations [2]. Tactical athletes likely stand to benefit from the use of sports nutrition supplements, such as caffeine and caffeine-like purine alkaloids, to improve these aspects of performance.

Caffeine has been studied extensively, and a recent systematic review concluded that caffeine in the range of 3–6 mg/kg relative to body mass has positive ergogenic effects on both physical and cognitive performance [3]. The primary mechanism of action by which caffeine exerts its ergogenic effects is central nervous system stimulation, primarily through adenosine receptor antagonism, attenuating feelings of drowsiness and fatigue [4]. Studies dating back to the 1930s have shown positive effects of caffeine on various measures of RT [5,6], and more recent work has corroborated these findings [7–13]. Overall, caffeine improves RT, movement time, and movement accuracy in healthy adults and athletes when consumed in moderate absolute doses of 300 mg or relative doses up to 6 mg/kg body mass, but higher doses of 600 mg or 7.5 mg/kg can be ergolytic [7–10]. In tactical populations, caffeine improves vigilance compared to placebo when consumed in doses ranging from 150 to 300 mg and has its largest effect when consumed in multiple consecutive doses during extended periods of sleep deprivation [11–13]. Marksmanship RT and accuracy are also improved by 200–300 mg caffeine [14–17], though one study noted greater feelings of hand trembling and irritability following 5 mg/kg and 2.5 mg/kg caffeine doses separated by 8 hours [17], both of which are commonly cited negative side effects of caffeine consumption [18–21].

Although caffeine is the most widely consumed and studied drug of the purine alkaloids, newly discovered compounds, such as methylliberine (Dynamine™) and theacrine (TeaCrine®) which are classified as tetramethylurates, exhibit structural similarities to caffeine and have similar pharmacodynamic effects. However, pharmacokinetic profiles of these compounds differ. Caffeine’s peak plasma concentration following oral consumption occurs within approximately 30–60 min with a half-life of approximately 4–5 hours [21]. Methylliberine has a shorter peak time of 0.6-0.9 hours with a half-life of 1.4 hours when consumed in doses of 25 mg and 100 mg [22], while theacrine has a longer peak time of about 1.8 hours and half-life of 16.5-26.1 hours when consumed in 25 mg to 125 mg doses [23]. Recent pharmacokinetic data for a combination of 100 mg methylliberine, 150 mg caffeine, and 50 mg theacrine showed peak plasma concentration times of 0.8, 1.1, and 1.4 hours with half-lives of 1.5, 21, and 30 hours, respectively [24]. This shows the potential synergistic effect of supplementation with these compounds as the differential peak times allow for the stimulatory actions to be maximized over a longer period rather than supplementing with one compound alone. Together, this can create a more sustained peak effect while also resulting in a more sustained time course of action.

Based on this theoretical rationale and demonstrated safety [23,25], the efficacy of a combination of caffeine with methylliberine and/or theacrine has been assessed in applied studies providing absolute rather than relative doses on various performance outcomes including RT [26–28]. Two studies showed no differences between caffeine-only conditions compared to combinations of caffeine and theacrine [26,27], while another showed the largest improvement in both RT and accuracy following 125 mg caffeine with 75 mg methylliberine and 50 mg theacrine compared to 125 mg caffeine [28]. Though preliminary, the results of these few studies show positive effects of combining caffeine with methylliberine and/or theacrine on cognitive tasks, RT, and accuracy, corroborating past research on the effects of caffeine on these outcomes.

One difference of particular interest between caffeine and these related compounds is the acute effect on hemodynamics. Generally, moderate-to-high doses of caffeine ranging from one cup of coffee (approximately 125 mg caffeine) to 300 mg caffeine acutely increase blood pressure and heart rate in normotensive individuals [29,30]. This effect also extends to exercise, as studies have shown greater increases in systolic (SBP) and diastolic blood pressure (DBP) during aerobic exercise bouts following consumption of ≥360 mg caffeine compared to placebo [31–33]. Based on these alterations observed following caffeine ingestion, resting hemodynamic responses following methylliberine and theacrine supplementation have also been investigated [25,34], and data show no changes in acute or chronic blood pressure responses following consumption in doses as high as 175 mg. However, when caffeine is included in combination with tetramethylurates, increases in hemodynamics appear to be attributed to caffeine content when compared to methylliberine or theacrine alone [28,35]. Law enforcement officers have been shown to experience increases in blood pressure throughout their career [36], therefore this is an outcome of particular interest in this population. Similarly, caffeine increase levels of physiological arousal [37–39], though felt arousal, a subjective measure, has not been shown to be increased by caffeine above placebo in males [40–43]. This response has not yet been assessed following tetramethylurate consumption.

Based on the known pharmacodynamics and pharmacokinetics of caffeine, methylliberine, and theacrine, it is speculated that co-ingestion of these compounds and sustained peak times and half-lives can improve physical and cognitive performances over a longer period compared to caffeine alone. Additionally, the more favorable hemodynamic responses along with data suggesting reduced jitteriness and habituation effects associated with methylliberine and theacrine [27,28,34] further support the idea of a beneficial effect of combining these supplements rather than consuming caffeine alone. Therefore, the purpose of this study was to compare the effects of a combination of caffeine, methylliberine, and theacrine to caffeine alone and placebo on RT and marksmanship following a sustained vigilance task along with hemodynamic responses and felt arousal in tactical personnel. It was hypothesized that the combination of these compounds would produce a synergistic effect, resulting in similar improvements in RT as caffeine alone above placebo, greater improvements in decision-making and accuracy compared to caffeine alone, and less pronounced hemodynamic responses and felt arousal compared to caffeine alone.

## 2. Methods

### 2.1. Experimental approach

A between-subjects, randomized, placebo-controlled design was used to test the effects of caffeine, methylliberine, and theacrine on RT and marksmanship along with hemodynamic and felt arousal measures following sustained vigilance. Participants were randomized into one of the three groups according to supplementation protocol: 300 mg cellulose placebo (PLA), 300 mg caffeine (CAF), or a combination of 150 mg caffeine, 100 mg methylliberine, and 50 mg theacrine (CMT). Following familiarization, participants arrived for the experimental testing session within 2 hours of waking, consumed the randomized supplement, and completed a 150-min protocol consisting of two rounds that each comprised vigilance, movement, and marksmanship tasks. Hemodynamics and felt arousal were assessed throughout the protocol corresponding to each task. Participants were instructed to abstain from caffeine for ≥24 h and alcohol for ≥48 h while maintaining normal sleep and wake schedules over the one-week period before the experimental testing session. All protocols and procedures were approved by the Rutgers University and University of South Carolina Institutional Review Boards. This study was registered on ClinicalTrials.gov (NCT03937687).

### 2.2. Participants

Forty-nine males currently employed as military personnel or law enforcement officers, currently enrolled in a Reserve Officers’ Training Corps (ROTC) program, or military veterans or retired law enforcement officers who had completed service within the past 18 months or were actively involved in tactical training or operations were recruited to participate in this study. All participants were between the ages of 18 and 63 years (inclusive) and had a body mass of at least 60 kg. Individuals were excluded if they had injuries that would prevent completion of the protocol, experienced migraines, had a history of hepatorenal or neurologic disease, had a history of caffeine sensitivity, were currently taking over-the-counter products containing pseudoephedrine or other stimulants, or were currently consuming ≥500 mg caffeine per day over the past year. All participants provided written informed consent.

### 2.3. Groups

Participants were randomized to one of the three groups: PLA, CAF, and CMT. All pills consisted of an encapsulated white powder and were identical across groups. The placebo capsule contained 300 mg cellulose, the caffeine capsule contained 300 mg caffeine, the combination capsule consisted of 150 mg caffeine, 100 mg methylliberine, and 50 mg theacrine. Thus, the total purine alkaloid content was matched at 300 mg for CAF and CMT, but the total caffeine content differed.

### 2.4. Body composition

Height was measured using a stadiometer, and body mass was measured using a calibrated scale. The body composition was assessed using air displacement plethysmography [BODPOD, COSMED Inc., Concord, CA, USA] [44,45];. Participants were instructed to arrive with non-padded compression shorts having refrained from food and water for ≥2 hours, caffeine for ≥12 hours, and moderate-to-vigorous physical activity or exercise for ≥24 hours. The device was calibrated, and each test was conducted according to manufacturer’s guidelines. A prediction equation was used to determine thoracic gas volume [46], and the Brozek model was used to determine body fat percentage from body density [47].

### 2.5. Vigilance task

The vigilance task consisted of a 30-min protocol using an interactive light board with 64 three-dimensional targets and a digital screen [D2, Dynavision International LLC, Cincinnati, OH, USA] [48];. Participants stood approximately 30 cm from the board, and the board was raised or lowered to a height ensuring the digital screen was at eye level and all lights could be reached. The blinds were drawn, and lights were turned off to reduce glare on the light board. A go/no-go task in which one of the 64 lights illuminated (either red or green) at a time was used, and participants were instructed to press the red lights as quickly as possible while avoiding the green lights. Each light remained illuminated for 2 seconds or until it was pressed, in which case another light illuminated. Simultaneously, every 9 seconds, the digital screen showed a three-integer arithmetic problem for 1 s to which participants were instructed to respond audibly to serve as a mental distraction before the next problem showed up. Researchers did not provide feedback during the task. Participants performed this task for 5 minutes during the familiarization session and 30 minutes during the experimental testing session. During the experimental testing session, RT for red lights and decision-making, quantified as a percentage of correct go/no-go decisions out of total decisions, were scored and recorded by the computer software.

### 2.5. Movement task

The movement task was a 40-target protocol using a computer simulator with full-body optical sensing technology [Trazer, TRAQ Global Ltd., Westlake, OH, USA] [49];. The simulator consists of a large digital screen and front-facing camera. The simulator was placed in front of an open space where a 3-meter-by-3-meter grid with a mark in the center was outlined on the floor using brightly colored tape. Participants stood on the mark in the center as the program was started, and the simulator self-calibrated for each assessment. The protocol consisted of targets appearing as pillars on the digital screen corresponding to locations 3 m in front, behind, left, or right of center, and participants were instructed to move to each target as quickly as possible and return to center after each target. Participants were required to reach 20 targets for each of the three trials during the familiarization session and 40 targets for each of the four trials during the experimental testing session. During the experimental testing session, RT was scored and recorded by the computer software.

### 2.6. Marksmanship task

The marksmanship task was a 16-target protocol using a shooting simulator (Smokeless Range, Laser Ammo, Great Neck, NY, USA) with a laser-modified, gas blowback airsoft pistol (ATP-C, KWA, City of Industry, CA, USA). This simulator has been used in previous research [50]. The simulator consists of a computer connected to a short-throw projector, along with a short-throw high-speed camera pointed at the projector screen which allows the software to determine where the laser-modified pistol was aimed when the trigger was pulled. The protocol consisted of digital law enforcement training targets (B-27, National Rifle Association, Fairfax, VA, USA) projected onto the screen one at a time, and participants were instructed to shoot each target as quickly and as accurately as possible. The round began with the participant holding the modified pistol at his hip, and participants were instructed to not lift the pistol until the first target appeared. In the middle of the round, a symbol came up on the screen indicating a tactical reload, in which participants released the magazine from the pistol and loaded the second magazine from a belt-mounted holster that was provided. Participants shot eight targets scaled to 15 m for each of the four trials, two of which consisted of a tactical reload, during the familiarization session and 16 targets scaled to 15 or 30 m for each trial during the experimental testing session. During the experimental testing session, RT was scored and recorded by the computer software, and accuracy, quantified as distance from center of target, was scored and recorded by a researcher using an image-processing program (ImageJ, National Institutes of Health, Bethesda, MD, USA).

### 2.7. Hemodynamic responses

**Figure 1.**
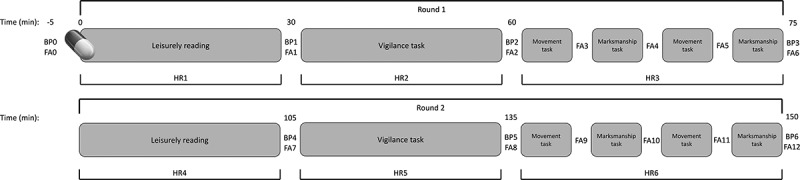
Overview of the experimental testing protocol. BP = blood pressure, FA = felt arousal, HR = heart rate.

SBP and DBP were measured using an automated blood pressure cuff [HEM 907XL, Omron Electronics LLC, Hoffman Estates, IL, USA] [51];. The participant was seated in the upright position with his arm supported at heart level by a table before a researcher placed the cuff snugly around the proximal portion of the right arm ensuring legs were not crossed. The participant was instructed to limit movement and refrain from talking during measurements. All blood pressure readings were measured in duplicate with 1 min between measurements. If measurements varied by >10%, a third measurement was taken, and the outlier measurement was removed. Blood pressure was measured at baseline before the participant consumed the capsule and at six timepoints throughout the protocol. These timepoints occurred before and following specific tasks throughout the protocol (Figure 1). Blood pressure readings were averaged across duplicate measurements at each timepoint.

Throughout the experimental protocol, heart rate was continuously monitored using a chest-strap heart rate sensor [H7, Polar Electro, Lake Success, NY, USA] [52]; connected via Bluetooth to a fitness watch (V800, Polar Electro, Lake Success, NY, USA). The watch was not worn by the participants but was kept near participants to ensure proper transmission between the sensor and the watch. Heart rate data were averaged across six blocks throughout the protocol.

### 2.8. Felt arousal

Felt arousal was measured using the Felt Arousal Scale [53], which ranges from 1 (low arousal) to 6 (high arousal). Felt arousal was assessed at baseline before the participant consumed the capsule and at 12 timepoints throughout the protocol.

### 2.9. Familiarization

Participants arrived at the Sport Science Laboratory having refrained from food and water for ≥2 hours, caffeine for ≥12 hours, and moderate-to-vigorous physical activity or exercise for ≥24 hours. Following screening and consenting, participants underwent body composition testing. Participants were then familiarized with the testing protocols including the vigilance, movement, and marksmanship tasks. Participants completed a shortened version of the vigilance task, three trials of the movement task, and four trials of the marksmanship task.

### 2.10. Experimental testing protocol

Prior to this session, participants were asked to maintain a normal sleep schedule in the week leading up to their session, maintaining the same wake time each day. Participants were also asked to refrain from caffeine for ≥24 hours and alcohol for ≥48 hours while maintaining habitual dietary habits. Instructions were provided to ensure participants arrived in an euhydrated state which emphasized consuming 2–4 L of water per day leading up to the visit. Prior to the start of the session, verbal confirmation of adherence to the pre-visit instructions was obtained. If participants did not adhere, the session was rescheduled. This session was scheduled to begin within 2 hours of waking and was within 30 min of the start time of the initial familiarization session. This 2-hour period was chosen to ensure consistency across participants since sleep and wake schedules can vary drastically in this population.

Participants arrived and sat quietly for 5 min. Following quiet rest, blood pressure was measured and felt arousal was assessed. Next, participants consumed the randomized capsule orally with water provided ad libitum. Participants then sat and read a book about exercise leisurely for 30 min. At the 25-min point of the 30 min quiet reading period, blood pressure and felt arousal were measured.

After 30 min of quiet reading was completed, participants began the 30-min vigilance task. Following completion of this task, participants returned to a seated position while blood pressure and felt arousal were again assessed. Immediately following these measurements, participants began the 40-target movement task, followed immediately by the 16-target marksmanship task with 15-m targets on the marksmanship simulator. This was again immediately followed by the same movement task and 16-target marksmanship task with 30-m targets. Felt arousal was assessed between each of these tasks. Upon completion of this first full round of testing at approximately the 75-min point, participants returned to a quiet seated position, and blood pressure and felt arousal were assessed. Participants then repeated the protocol in its entirety. An overview of the experimental testing protocol is shown in Figure 1.

### 2.11. Data analysis

One-way analyses of variance were conducted to determine differences in baseline descriptive metrics between groups, and chi-square tests were used to determine differences in felt arousal and participant occupations between groups. For vigilance, movement, and marksmanship task variables, RT, cognitive decision-making, and accuracy scores were analyzed. For composite vigilance and marksmanship task performance, Z-scores were computed for RT and cognitive decision-making (vigilance) or accuracy (marksmanship) to compare performance relative to the cohort mean at each respective timepoint. The inverse of RT Z-score was calculated, and the product of RT and cognitive decision-making or accuracy Z-scores was used for composite score analyses. Linear mixed-effects models with a random intercept to adjust for between-subject variability were used to test for Group (PLA, CAF, and CMT)-by-Time (two rounds) interactions, as well as main effects of Group and Time. For hemodynamic variables, linear mixed-effects models with a random intercept to adjust for between-subject variability were used to test for Group (PLA, CAF, and CMT)-by-Time (seven timepoints for SBP and DBP, six timepoints for HR), as well as the main effects of Group and Time. For felt arousal, a generalized linear mixed-effects model with a random intercept to adjust for between-subject variability was used to test for a Group (PLA, CAF, and CMT)-by-Time (13 timepoints) interaction, as well as the main effects of Group and Time. Significant interactions or main effects were followed up with post-hoc pairwise comparisons with a Bonferroni adjustment to further explain the effects. An α-level of 0.05 was used to determine the statistical significance. Effect sizes were calculated using Cohen’s *d* to determine the magnitude of change within groups and differences between groups. All analyses were conducted and figures were produced using commercially available open-source statistical software [R; version 4.1.0] [54]; with the lme4 [version 1.1-27.1] [55]; emmeans [version 1.6.2-1] [56]; effectsize [version 0.5] [57]; and ggplot2 [version 3.3.5] [58]; packages.

## 3. Results

### 3.1. Participants

One participant was lost to follow-up, so the total number of participants that completed the protocol was 48. The participants consisted of 35% law enforcement officers, 19% military personnel and veterans, and 46% ROTC cadets and midshipmen. A chi-square test revealed no differences in participant occupation between groups (χ^2^_4_ = 1.25, P = 0.868). One participant in CAF was not included in the analysis of marksmanship measures due to equipment technical malfunctions. One participant in the PLA group was not included in the analysis of hemodynamic measures due to equipment technical malfunctions.

### 3.2. Baseline characteristics

Descriptive baseline statistics are shown in Table 1. There were no significant differences between groups in baseline metrics.

**Table 1.** Baseline descriptive characteristics by group.

Group	PLA (n = 17)	CAF (n = 16)	CMT (n = 15)	P-value
Age (y)	25.8 ± 9.8	25.8 ± 7.2	30.8 ± 13.2	0.296
Experience (y)	4.9 ± 8.6	4.8 ± 6.5	8.9 ± 10.4	0.323
Height (cm)	182 ± 6	178 ± 6	179 ± 6	0.103
Body mass (kg)	85.1 ± 20.2	82.2 ± 15.0	85.0 ± 8.8	0.089
Body fat percentage	15.6 ± 7.1	16.9 ± 7.7	18.5 ± 4.8	0.479
Caffeine intake per day (mg)	116 ± 150	162 ± 131	80 ± 88	0.211
Caffeine intake per day (mg/kg)	1.3 ± 1.6	1.9 ± 1.6	0.9 ± 1.0	0.170
SBP (mmHg)	117 ± 12	126 ± 12	125 ± 8	0.057
DBP (mmHg)	70 ± 10	72 ± 11	68 ± 10	0.504
Felt arousal (au)	2 ± 1	2 ± 1	3 ± 1	0.298

Data are presented as mean ± SD. P-value shows result of one-way ANOVAs or chi-square test.

### 3.2. Vigilance task

**Figure 2.**
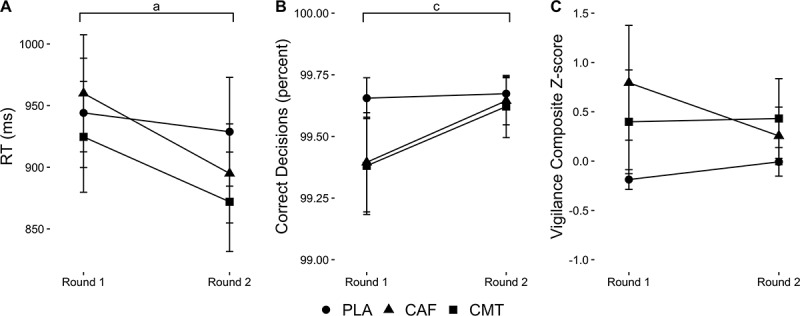
Changes in (A) RT, (B) correct decisions, and (C) composite Z-score relative to PLA from round 1 to round 2 of the experimental testing protocol between groups during the vigilance task. ‘a’ denotes a Group-by-Time interaction; ‘c’ denotes a Time main effect.

Analyses revealed a Group-by-Time interaction for RT (F_2,45_ = 3.52, P = 0.038), Time main effect for decision-making (F_1,45_ = 5.74, P = 0.021), and no effects for composite Z-score (P > 0.19). For RT, post-hoc tests showed significant improvements from round 1 to round 2 in CAF (P < 0.01; *d*= −0.37) and CMT (P < 0.01, *d*= −0.32), but not PLA (P = 0.27, *d*= −0.08). Regardless of group, decision-making improved from round 1 to round 2 (PLA: *d*= 0.06, CAF: *d*= 0.39, CMT: *d*= 0.38). Data are shown in Figures 2A-C.

### 3.3. Movement task

**Figure 3.**
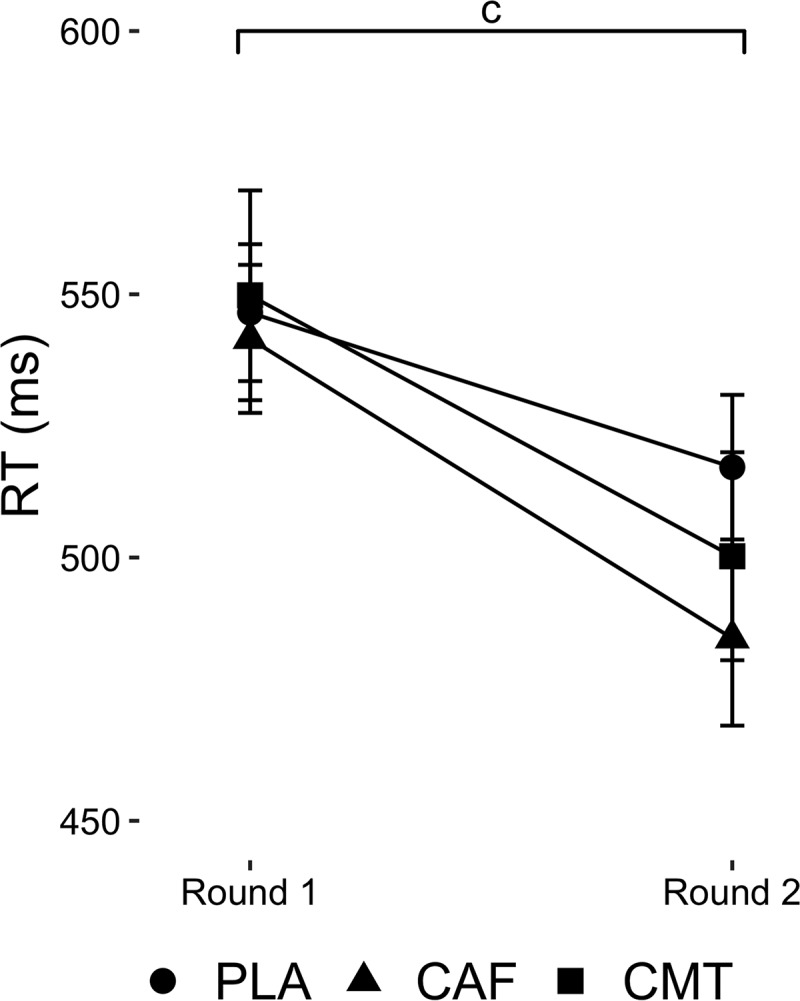
Changes in RT from round 1 to round 2 of the experimental testing protocol between groups during the movement task. ‘c’ denotes a Time main effect.

No Group-by-Time interaction or Group main effect was found for RT (P > 0.22). A time main effect was observed for RT (F_1,141_ = 44.17, P < 0.001). Regardless of group, RT improved from round 1 to round 2 for all groups (PLA: *d*= 0.38, CAF: *d*= 0.65, CMT: *d*= 0.46). Data are shown in Figure 3.

### 3.4. Marksmanship task

**Figure 4.**
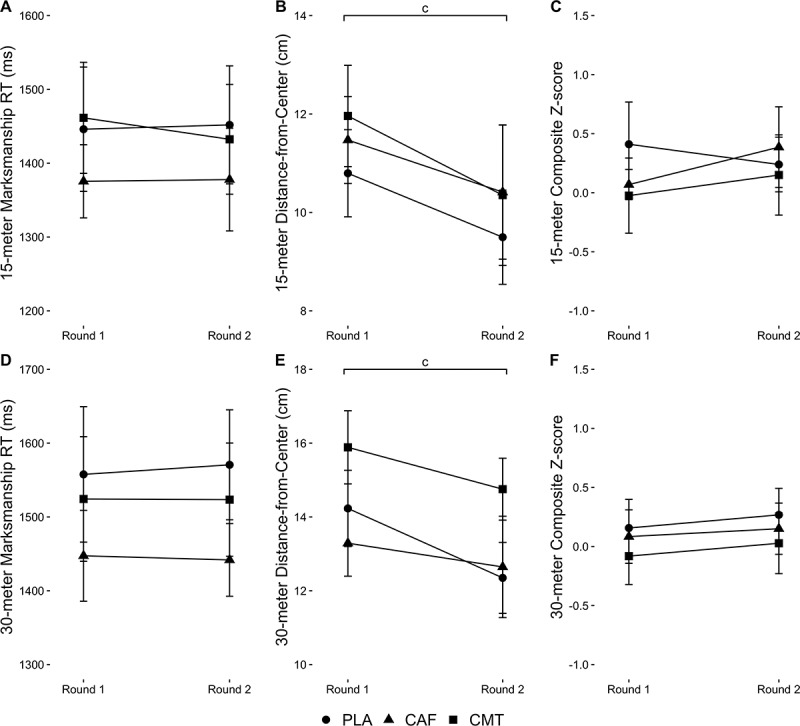
Changes in (A) 15-meter RT, (B) 15-meter accuracy, (C) 15-meter composite Z-score, (D) 15-meter RT, (E) 15-meter accuracy, and (F) 15-meter composite Z-score from round 1 to round 2 of the experimental testing protocol between groups during the marksmanship task. ‘c’ denotes a Time main effect.

No Group-by-Time interactions or Group main effects were found for RT, accuracy, or composite Z-scores during the marksmanship task (P > 0.20). Time main effects were observed for accuracy during the 15-m (F_1,24.7_ = 18.83, P < 0.001) and 30-m trials (F_1,33.9_ = 6.00, P = 0.020). Regardless of group, accuracy improved from round 1 to round 2 for the 15-m (PLA: *d*= −0.41, CAF: *d*= −0.27, CMT: *d*= −0.39) and 30-m trials (PLA: *d*= −0.50, CAF: *d*= −0.14, CMT: *d*= −0.38). Data are shown in Figures 4A-F.

### 3.5. Hemodynamic responses

**Figure 5.**
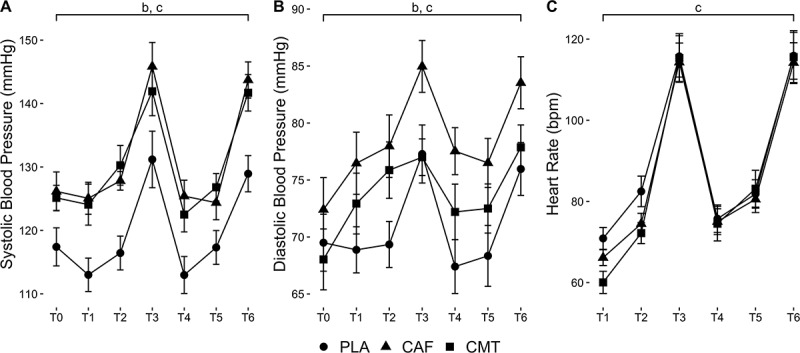
Changes in (A) SBP, (B) DBP, and (C) heart rate throughout the experimental testing protocol between groups. ‘b’ denotes a Group main effect; ‘c’ denotes a Time main effect.

Group and Time main effects were found for SBP (F_2,44_ = 7.87, P = 0.001; F_6,264_ = 54.32, P < 0.001) and DBP (F_2,44_ = 3.89, P = 0.028; F_6,264_ = 18.55, P < 0.001). For SBP, post-hoc tests showed higher values in both CAF (P = 0.003, *d*= 0.84) and CMT (P = 0.007, *d*= 0.79) compared to PLA, with no differences between CAF and CMT (P = 1.00, *d*= 0.06). For DBP, post-hoc tests showed higher values for CAF compared to PLA (P = 0.025, *d*= 0.74), with no differences between CMT and PLA (P = 0.947, *d*= 0.29) or CAF and CMT (P = 0.288, *d*= 0.48). Regardless of group, SBP was higher than baseline at T3 (P < 0.001) and T6 (P < 0.001), and DBP was higher than baseline at T2 (P = 0.002), T3 (P < 0.001), and T6 (P < 0.001). Heart rate responses exhibited a time main effect (F_5,156.8_ = 247.7, P < 0.001). Post-hoc tests revealed increases from T1 at all subsequent time points (P < 0.001). Data are shown in Figures 5A-C.

### 3.6. Felt arousal

**Figure 6.**
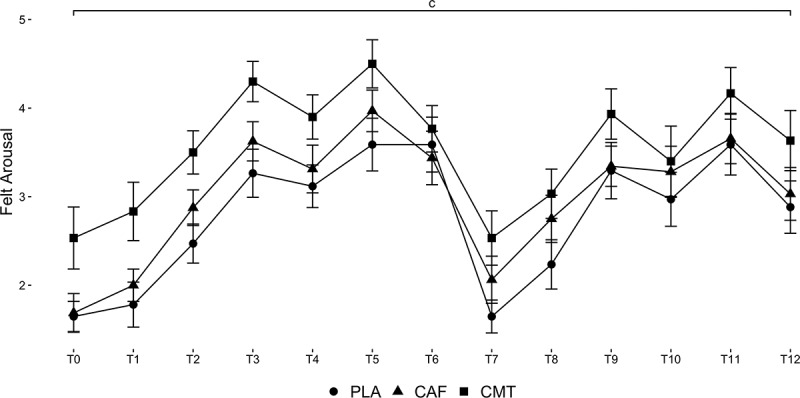
Changes in felt arousal throughout the experimental testing protocol between groups. ‘c’ denotes a Time main effect.

A Time main effect was found for felt arousal (χ^2^_12_ = 40.54, P < 0.01). Regardless of group, felt arousal was higher than baseline at all timepoints (P < 0.001) except T1, T7, and T8 (P > 0.15). Data are shown in Figure 6.

## 4. Discussion

This is the first study to test the effects of caffeine in conjunction with methylliberine and theacrine on vigilance and marksmanship. The primary findings show a combination of 150 mg caffeine, 100 mg methylliberine, and 50 mg theacrine improves RT during a vigilance task from round 1 to round 2 to a similar extent as 300 mg caffeine alone while placebo does not. These findings support the hypothesis that CMT would exhibit improved RT similar to CAF when compared to PLA. However, it was also found that CMT did not improve decision-making or accuracy during vigilance or marksmanship tasks above caffeine alone or placebo, which does not support the hypothesis based on previous data suggesting less jitteriness as well as more sustained peak plasma concentrations associated with the combination of caffeine, methylliberine, and theacrine compared to a single dose of 300 mg caffeine. Over the same time course, CMT and CAF both resulted in increases of large effect sizes in SBP above PLA. Caffeine alone, however, resulted in large elevations in DBP compared to placebo while the combination of caffeine, methylliberine, and theacrine did not. Though CAF was not statistically different from CMT, the difference in magnitude suggests a more favorable hemodynamic response in CMT compared to CAF as higher exercising blood pressures may contribute to cardiovascular death and myocardial infarction [59,60]. These data partially support the hypothesis that CMT would exhibit less severe perturbations in blood pressure than CAF. Further, the results showed no differences in movement RT between groups supporting the hypothesis that CMT and CAF would be similar, although lack of improvements in these groups above PLA do not corroborate previous research. Similarly, the lack of differences in marksmanship performance across groups do not support the hypothesis that CAF but not CMT would result in decrements compared to PLA. Finally, felt arousal was similar across all groups throughout the protocol, which does not support the hypothesis of a less pronounced response in CMT compared to CAF.

Maintaining vigilance is critical during sustained operations in tactical populations, and caffeine has consistently shown favorable effects on this task compared to placebo [11–13,61]. Although research designs vary greatly across studies on this topic in terms of caffeine dose, protocols, and outcome measures, this effect is consistent and is supported by the data presented here. The absolute dose of 300 mg or relative dose of 3.56 mg/kg based on the mean body mass in the sample of total caffeine or combined content provided in the current study would be considered a moderate, efficacious dose [3]. A similar study by Kamimori et al. [11] compared 2.1, 4.3, and 8.6 mg/kg caffeine to placebo on measures of choice RT during vigilance following 48 hours of sleep deprivation and found all caffeine conditions resulted in improved RT over placebo. Further, McLellan et al. [12] provided multiple 200-mg caffeine doses during an overnight vigilance task which required soldiers to record all activities that occurred in an illuminated building situated 175–200 meters away for 120 min in 20 special operators and showed maintenance of vigilance in the caffeine condition while placebo resulted in decrements. The results presented here indicated no differences between the combination and caffeine only, suggesting the addition of methylliberine and theacrine provide similar ergogenic effects as caffeine alone perhaps due to the equivalent purine alkaloid content between groups.

Marksmanship performance, another crucial skill for the tactical athlete during and following periods of sustained vigilance, was unaffected by CMT or CAF compared to PLA. A recent systematic review concluded that marksmanship accuracy is improved by caffeine doses of 100–200 mg consumed every 2 hours [62], suggesting the 300 mg dose provided in the current study may have been too large to improve marksmanship. However, a study conducted by Gillingham et al. [16] in military reservists showed no differences in marksmanship performance between 300 mg caffeine and placebo conditions during periods of vigilance following sleep deprivation. This corresponds to the findings here and could be considered as a positive effect as caffeine consumption is often associated with jitteriness [18–21], but no negative effects on marksmanship performance were observed. Interestingly, the lower 150 mg dose of caffeine combined with methylliberine and theacrine did not result in improved marksmanship above 300 mg caffeine alone despite the lack of jitters associated with this combination [27,28], though this was not different from PLA. Additionally, RT measured during the movement task in this study also did not result in improvements over placebo following caffeine consumption, which is contrary to much of the existing literature.

The improvement in vigilance above placebo and maintenance of marksmanship accuracy observed in both CMT and CAF is even more substantial when considering the hemodynamic responses observed in the current study. It is commonly cited that moderate doses of caffeine increase blood pressure at rest [29,30] and during exercise [31–33]. This increase was observed in both supplement groups with regard to SBP, but DBP was greater throughout the entire 150-min protocol in CAF compared to PLA with a moderate-to-large effect size but was not elevated above PLA in CMT. This finding is consistent with previous work as VanDusseldorp et al. [25] observed no changes in acute or chronic blood pressure responses following supplementation with methylliberine alone, theacrine alone, or a combination of methylliberine and theacrine. Similarly, Bloomer et al. [35] recently showed increases in both SBP and DBP following consumption of methylliberine and theacrine only when consumed with caffeine. Although this slightly contradicts the current finding that SBP but not DBP was elevated above placebo, it shows that caffeine appears to be the driver in altered hemodynamics at rest and exercise and a lower dose of caffeine results in lesser perturbations in hemodynamics. The observation of no differences in heart rate across groups is consistent with previous research as caffeine does not consistently affect this measure [30].

Felt or perceived arousal was not different between any of the groups throughout the 150-min protocol. This finding is consistent with previous studies showing no effects of caffeine consumption ranging from 5 to 6 mg/kg on ratings of felt arousal compared to placebo in sample sizes of 8 to 15 young males during and following both resistance and endurance training bouts [40–43]. This outcome, however, has not been assessed previously in studies providing methylliberine or theacrine. Therefore, this is the first study to show no further increases in physiological activation following consumption of caffeine, methylliberine, and theacrine compared to caffeine alone or placebo.

Overall, these findings show consistency with previous research, and there are several strengths, limitations, and delimitations to the current study. One strength is the protocol used as it mimics military and law enforcement tactics. The vigilance task simulated an overwatch scenario as it required participants to maintain mental acuity by using peripheral vision to see lights illuminate on the board and decide whether to press the button based on the color of the light while simultaneously remaining focused on the digital screening and performing mental math. Next, participants continued to the movement task which required physical movement resulting in elevations in heart rate and blood pressure before moving into the marksmanship task with a tactical reload. These tasks mimic undergoing a foot pursuit or physical confrontation, identifying key activities and friend versus foe, and then engaging in a firefight requiring accuracy and speed of decision-making. Additionally, the use of 300 mg purine alkaloids is a strength to the design as these supplements are often consumed through capsules and beverages in absolute rather than relative doses. Further, previous research on habitual caffeine use in law enforcement and military populations has indicated intakes of approximately 250 mg/day [63,64], suggesting this is an appropriate and efficacious dose.

One limitation, however, was that participants were recruited from law enforcement, military, and ROTC. Marksmanship training is likely different across these populations in terms of weapon preference and years of shooting experience, and there are considerable differences in skill and expertise within each of these populations as well. To address this, all participants experienced the same familiarization protocol prior to the experimental testing. It is important to note that these categories of participants were evenly distributed across experimental groups. Additionally, the marksmanship simulator and airsoft pistol used in this study do not match outdoor, live-fire conditions used in previous research, but the immediate feedback provided through the laser and high-speed camera as well as gas blowback of the pistol provided simulated conditions. A delimitation is that these findings do not necessarily extend to female tactical personnel. Based on inherent differences in RT between males and females [65] as well as the fact that females make up less than 15% of law enforcement officers and active-duty military personnel, females were excluded from participating in this study. Further, another delimitation that reduces consistency with previous research on tactical outcomes is the lack of a sleep deprivation protocol prior to supplementation. This was not included in this study as law enforcement officers and military personnel do not always operate under sleep deprived conditions, and the purpose of the study was to determine the effects of the supplements provided under typical operating conditions. Therefore, participants were instructed to maintain normal sleep and wake schedules during the week leading up to the experimental testing session.

Together, the unique design of this study provides compelling evidence for the efficacy of combining a lower dose of caffeine with methylliberine and theacrine for maintaining vigilance to a similar extent as caffeine alone and maintaining marksmanship without largely increasing DBP. Further, despite its distinct protocol compared to other literature on this topic, this study corresponds with previous findings regarding caffeine on measures of RT, vigilance, and marksmanship and on a combination of caffeine, methylliberine, and theacrine on hemodynamics during exercise. Finally, this study is the first to test the effects of this supplementation protocol on tactical performance outcomes and to report felt arousal responses following consumption of a combination of caffeine, methylliberine, and theacrine.

## 5. Conclusions

Both CAF and CMT improved vigilance RT over the 150-min protocol while PLA did not, albeit no differences were observed in vigilance composite score between groups. CAF resulted in large increases in DBP above PLA while CMT did not, though CAF and CMT were not different from each other. Overall, these findings suggest similar overall effects on tactical performance measures between 300 mg caffeine alone and a combination of 150 mg caffeine, 100 mg methylliberine, and 50 mg theacrine along with slightly more favorable hemodynamic responses following supplementation with the combination compared to caffeine alone. Tactical athletes including law enforcement officers and military personnel can use this supplementation strategy of combined caffeine, methylliberine, and theacrine prior to periods of overwatch to maintain vigilance without experiencing substantial adverse hemodynamic responses induced by caffeine alone.

## Data Availability

The data collected and analyzed in this study are available from the corresponding author upon reasonable request.

## List of Abbreviations

RT: reaction time; SBP: systolic blood pressure; DBP: diastolic blood pressure; PLA: placebo; CAF: 300 mg caffeine; CMT: 150 mg caffeine, 100 mg methylliberine, 50 mg theacrine; SBP: systolic blood pressure; DBP: diastolic blood pressure; ROTC: Reserve Officers’ Training Corps

## Declarations

This study was registered on ClinicalTrials.gov (NCT03937687) on May 6, 2019.
